# Polymer Sorting Through Fluorescence Spectra

**DOI:** 10.3390/bioengineering12070708

**Published:** 2025-06-28

**Authors:** C. M. Penso, Elisabete M. S. Castanheira, Maria C. Paiva, L. M. Gonçalves

**Affiliations:** 1 Centre for MicroElectromechanical Systems (CMEMS-UMinho), University of Minho, 4800-058 Guimarães, Portugal; lgoncalves@dei.uminho.pt; 2 Centre of Physics of Minho and Porto Universities (CF-UM-UP), Campus de Gualtar, University of Minho, 4710-057 Braga, Portugal; ecoutinho@fisica.uminho.pt; 3 Departamento de Engenharia de Polímeros, Instituto de Polímeros e Compósitos, University of Minho, 4800-058 Guimarães, Portugal; mcpaiva@dep.uminho.pt; 4 LABBELS–Associate Laboratory, 4710-057 Braga, Portugal

**Keywords:** optical sensor, fluorescence, polymer, plastic pollution, microplastics

## Abstract

This study identifies different polymers using their fluorescent data under various light wavelengths that ranged from 245 nm to 345 nm in 10 nm intervals. The primary goal of the proposed method is to select optimal wavelengths that can lead to the accurate identification of six polymers: polyamide 6 (PA6), polymethyl methacrylate (PMMA), polypropylene (PP), polystyrene (PS), high-density polyethylene (HDPE), and polyethylene terephthalate (PET). By examining the specific fluorescence emission patterns of these polymers, the study provides insight into how each material responds uniquely to different excitation light sources. The potential approach could streamline polymer identification in recycling applications or even in quality control and environmental monitoring, including microplastics. This approach could lead to improved accuracy in polymer classification, contributing to more efficient material sorting and processing.

## 1. Introduction

The ever-growing applications of polymers have created great advancements in various fields, ranging from medicine to technology and consumer goods [1]. Plastics have been used to make products that have transformed the industry with their versatility, durability, and low cost. Over the years, however, the increasing pollution caused by polymers has been something that we have kept our eyes blinded to [2]. When thrown away improperly, these residues end up dumped in the oceans and soil. This creates contamination in food chains that poses a threat to both wildlife [3,4,5] and human health [6]. Microplastics are one of the smallest plastic entities that represent a tremendous challenge since they are ubiquitous in all sorts of environments—from the busiest cities to the last wild ecosystems, such as deep ocean floors and the Arctic [7,8]. This global spread highlights the ability of microplastics to disperse and integrate into ecological chains, raising significant concerns about their environmental and public health impacts. The most common polymers found in our daily lives include polyethylene (PE), used in plastic bags and bottles; polypropylene (PP), present in packaging, containers, and utensils; polyethylene terephthalate (PET), widely used in beverage bottles and food packaging; polystyrene (PS), found in disposable products like cups and plates; and polyvinyl chloride (PVC), used in piping, cable coatings, and construction materials. A great portion of polymers released into the environment eventually undergo slow photodegradation into smaller pieces as a result of sunlight, microbial activity, and mechanical abrasion [9]. The smaller these plastics become, turning into microplastics and even nanoplastics, the harder it is to detect and analyze them. The characteristics and properties of smaller particles, due to their higher surface area to volume ratio, become very different from those of larger particles, which makes their identification very complex. Furthermore, these fragments are much more readily transported through the air, water, and soil, which makes it very difficult to remove these substances from the environment, thereby causing long-term health and ecological problems and concerns [10].

The most popular plastics identification techniques include Fourier-transform infrared spectroscopy (FTIR) and Raman spectroscopy [11]. FTIR techniques utilize the absorption of infrared radiation to ascertain the chemical makeup of particular plastics. Alternatively, Raman spectroscopy gives information regarding the molecular structure of samples by means of inelastic light scattering. FTIR is well-suited for most of the chemical identification of microplastics, particularly for the larger ones (generally greater than 20 μm), as it offers absorption spectra that contain detailed information that enables the determination of polymer types. However, FTIR is less effective in the analysis of smaller particles and has the additional limitation of being unable to report non-organic constituents; in other words, clean samples devoid of water contamination are indispensable [12]. Raman spectroscopy, on the other hand, is superior in its examination of small microplastics, even to the order of nanometers, and can operate in water without much damage from water. Furthermore, Raman detection can be used for organic and inorganic substances. Its drawbacks include fluorescence interference, which can wedge the spectra, and comparatively low speed compared to FTIR in acquiring the data. Both Raman spectroscopy and FTIR spectrometry prove invaluable for the compositional analysis of microplastics. However, the technique of capturing, filtering, and isolating microplastic particles is extremely tedious and difficult, often resulting in contamination from the cleaning process or particles being unintentionally removed, which leads to inaccurate counts of the quantity of particles [13]. This emphasizes the increasing demand for more economical and expedited analysis techniques that would improve the efficiency of microplastic detection and measurement.

Some research has attempted to use fluorescence with particular dyes for polymer recognition, in which case, dyes attach to plastic particles and, under certain wavelengths, emit specific fluorescence. This approach has shown potential for improving the sensitivity and precision of microplastics detection, particularly in difficult samples. The dyes most frequently used for the identification of polymers are Nile Red, Rhodamine B, Safranin T, and Eosin B [14]. However, aside from the flaw of having to rely on a dye, a more serious problem with this method is that the range of fluorescence emission for most dyes lies within 540–640 nm (depending on the dye), where organic matter in the sample substrate (or even biofilms that have formed on the surface of the polymer) possess some degree of fluorescence. This phenomenon can mask the accurate identification of polymers. Also, while applying dyes for staining the polymers, some materials give out this signal too weak to be picked for measurement. In more complex cases, like fibers, staining can be non-uniform. Many of these dyes are poisonous and may aggravate pre-existing environmental pollution [14].

In oceanic contexts, the natural organic matter fluorescence is greatest in the visible range of 400 to 500 nm, which encompasses the blue and green wavelengths. This is primarily attributed to humic substances, which form a large part of dissolved organic matter, along with phytoplanktonic biological degradation products. Nonetheless, the application of dyes in fluorescence studies is often misleading because most dyes pass through the 400 to 670 nm band, which a priori overlaps with the natural fluorescence band of organic matter. This overlap can create interference and makes it difficult to separate real signals from artificial signals produced by dye sources. Beyond the use of dyes, polymers can also exhibit intrinsic fluorescence, allowing their identification based on their natural spectral footprint and fluorescence lifetime [15]. By analyzing these properties without the need for external dyes, it is possible to differentiate polymers through their unique fluorescence emissions and decay times (around 0.5–10 ns), offering a non-invasive and potentially faster approach for polymer identification.

The fluorescence lifetime measurement technique typically requires more sophisticated and expensive equipment (since the lifetime is typically in the range of nanoseconds). Fluorescence lifetime analysis involves complex instrumentation, and the data processing itself is algorithmically challenging. This makes the method less accessible and less practical for large-scale or field applications. The application of dyes and impurities is less noticeable when it comes to the sensitivity of UV fluorescence techniques. This insensitivity comes from the fact that many stains and contaminants do not absorb and emit energy in the form of light in the ultraviolet region, which reduces interference in the measurements taken. This means that ultrasound scanning with fluorescence can be performed with more accuracy and reliability in the presence of contaminants, as the effect on the signal is largely imperceptible [16].

This work introduces a novel lab-on-a-chip concept for polymer identification, based on fluorescence detection using UV-LED excitation, spectrally filtered photodetectors, and classification algorithms. The proposed approach offers a compact, low-cost, and scalable alternative to conventional spectroscopic methods, with promising applications in environmental monitoring and biomedical analysis.

## 2. Materials and Methods

Six different plastic types as pellets, PMMA Altuglas GR 7E from ARKEMA, PS from Styrolution 158 K (crystal clear) from INEOS, HDPE Eraclene MP90 U from VERSALIS, PA6 Badamid B70 from BADA AG, PP ISPLEN PP080G2M from REPSOL, and PET injection molding grade from SELENIS, were used.

A thermal press was employed to transform the pellets into thin films with a thickness ranging from approximately 0.004 to 0.006 mm. The resulting films are transparent. The melting points and thermal press temperatures used for each polymer are summarized in Table 1.

Fluorescence spectra of the films were analyzed within the range of 250 to 480 nm (1 nm resolution), with excitation wavelengths spanning from 245 nm to 355 nm (10 nm resolution). These measurements were conducted using a Fluorolog 3 spectrofluorometer (Horiba—Jobin Yvon, Palaiseau, France), equipped with double monochromators in both excitation and emission.

### 2.1. Device Proposal

**Figure 1:**
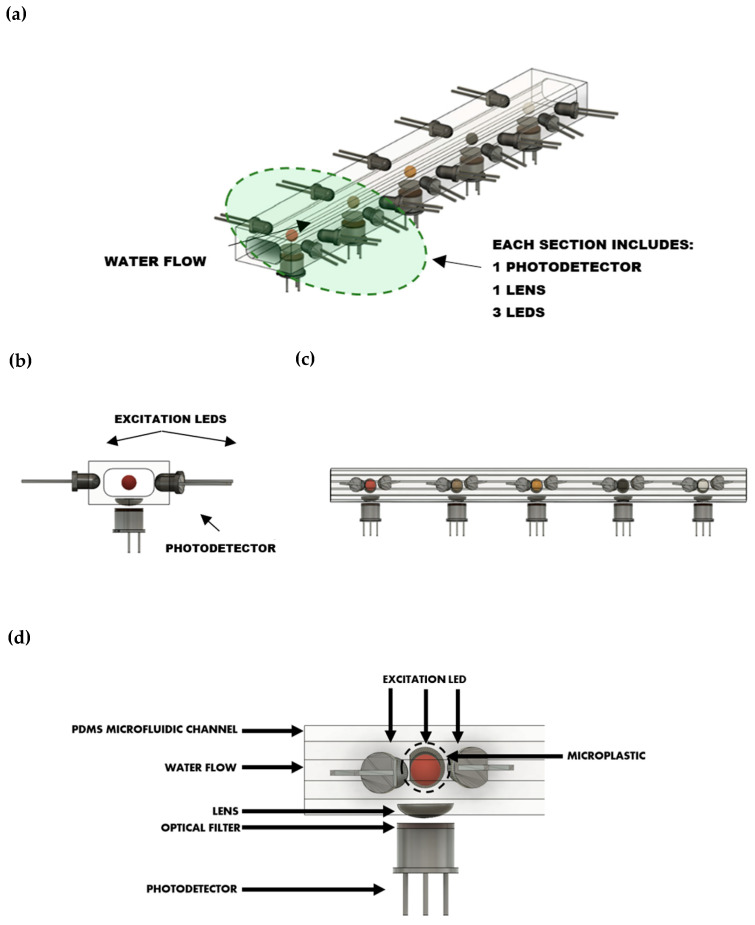
Sensor setup design, including a lab-on-chip for particle alignment and 5 detection sections. Each section includes a photodetector and 3 excitation LEDs: (**a**) isometric view of complete device; (**b**) cross-sectional view of each section; (**c**) lateral view of complete device; (**d**) detailed lateral view of each section.

A lab-on-chip device is designed for the fluorescent detection of microplastics, as illustrated in Figure 1. The sensor configuration includes a lab-on-chip that channels the plastic samples into a microfluidic pathway, analogous to flow cytometry. Every plastic fragment is exposed to UV light from LEDs of different wavelengths as it moves through the channel. Specific UV excitation induces fluorescence, which is measured by photodetectors. A non-destructive measurement of fluorescence is made possible, along with the measurement of different types of plastics with different fluorescent signatures.

The sensor setup (Figure 1) is composed of 5 identical sections. Each section analyses fluorescence emission in a selected wavelength (in the range 305–455 nm). Each section comprises three UV LEDs (265 nm, 285 nm, and 355 nm) positioned in an arc on a support, with each directed toward the center of the PDMS-based microfluidic channel through which water containing plastic microparticles flows. The purpose of this configuration is to ensure that the radiation from each LED reaches the particles but not the photodetector. The photodetector is positioned in a direction at 90°. This alignment allows the photodetectors to capture and analyze the radiation of fluorescence, without receiving direct light from the LED.

### 2.2. Optical Detection System

The sensor’s optical system is configured to capture and focus the fluorescence emissions efficiently. Three LEDs, operating at 265 nm, 285 nm, and 355 nm, provide controlled excitation light to the sample. The emitted fluorescence is captured by photodetectors with wavelength filters for five nominal channel wavelengths: 305, 355, 370, 405, and 455 nm. These filters dramatically aid in weak stray light and background signal detection by helping isolate only the pertinent spectral bands. Further, each filter is combined with a converging lens that captures and focuses from the sample onto the photodetector to enhance signal collection, improving detection sensitivity within the desired spectral region. The collection optics must be carefully implemented to collect as much signal as possible and cut out signal losses to ensure strong sensitivity and accuracy when identifying different plastic material fluorescence. The components proposed for the optical setup are listed in Table 2.

### 2.3. Readout Electronics

The sensor’s electronic system is designed to control three LEDs, each emitting at a distinct excitation wavelength, and to capture fluorescence responses across five different detection wavelengths. A set of photodetectors measures the emitted fluorescence from each sample after LED excitation. A transimpedance amplifier is proposed to convert the current of the photodetector (photodiode) to voltage. The data acquisition is synchronized through microcontroller-based electronics, ensuring precise timing and control over the excitation and detection processes. The software, developed to process the fluorescence signals, computes intensity ratios from the measured data (see Table 3). For each excitation wavelength, the five readouts from the photodetectors are compared, calculating the ratio between them. As an example, the first line in Table 3 (where 265 nm excitation was used) shows the ratio of emission intensity measured at 305 nm and 355 nm for the 6 polymers studied. Readouts at wavelengths below the excitation wavelength were not considered; fluorescence always occurs at wavelengths above the excitation.

## 3. Results

The primary aim of this study was to identify optical properties that can differentiate between six types of polymers (PMMA, PS, PVC, HDPE, PP, and PA6).

### 3.1. Fluorescence Spectra

**Figure 2:**
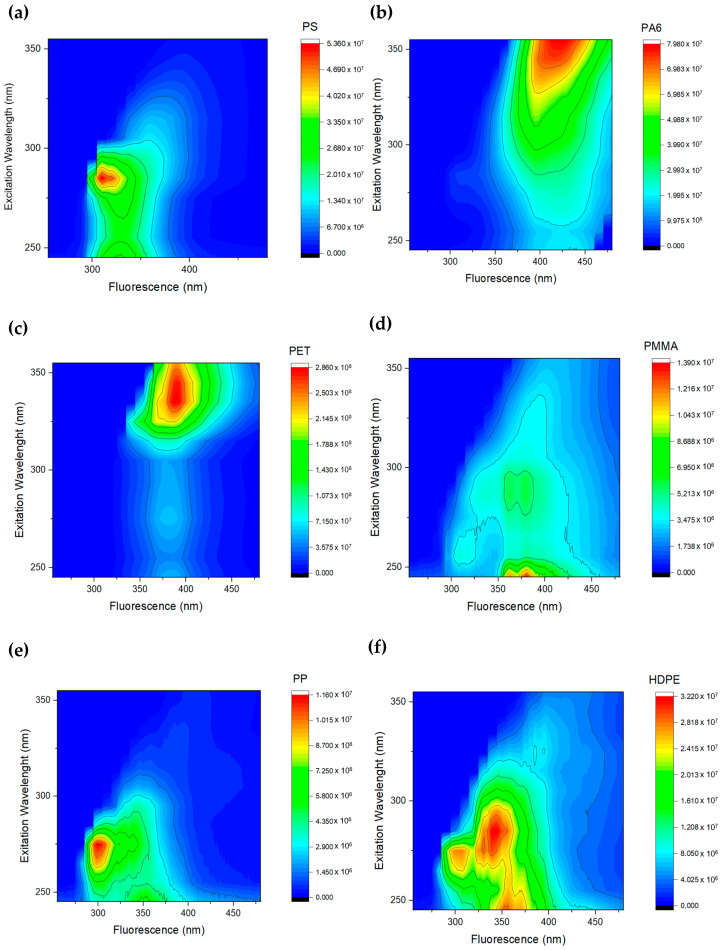
EEM matrices for various polymers. Each subfigure (**a**–**f**) represents a different polymer in the following order: (**a**) PS, (**b**) PA6, (**c**) PET, (**d**) PMMA, (**e**) PP, and (**f**) HDPE.

Figure 2 represents an excitation–emission matrix (EEM) plot. In these plots, the y-axis indicates the excitation wavelength, while the x-axis represents the emission wavelength. The intensity of fluorescence is depicted by a color gradient from blue to red, where lower intensities of fluorescence are lighter blue while higher intensities of fluorescence are warmer colors like green, yellow, and orange, as illustrated on the scales in Figure 2.

The fluorescence spectra of various polymers, obtained at different excitation wavelengths for each polymer, reveal distinct fluorescence peaks for each excitation. These unique spectral features vary across different polymers, allowing for their differentiation through spectral analysis.

In a visual analysis of Figure 2, the polymers PS, PP, and HDPE have fluorescence peaks with emission in the range 300–350 nm, when excited in the range 260–300 nm, in contrast to PET, PA6, and HDPE. The PS excitation wavelength is slightly higher (285 nm) than PP (270 nm). HDPE has a higher emission wavelength (peak at 350 nm) than the former PS and PP.

Both PET and PA6 are the only ones that can be excited at 330–350 nm, but emission peaks are at 380–400 nm for PET and 400–430 nm for PA6. PMMA presents the fluorescence emission peak at 350–400 nm, excited with wavelengths below 250 nm.

Despite the differences in fluorescence spectrum of these plastics, a device to measure all the spectra as presented in Figure 2 (where emission was measured with 1 nm resolution with high-sensitivity detectors) would be bulky and expensive. Based on the analysis of Figure 2, the UV LEDs and optical filters availability, the excitation wavelengths of 265 nm, 285 nm, and 355 nm, and the emission wavelengths of 300 nm, 355 nm, 370 nm, 405 nm, and 455 nm were chosen.

### 3.2. Classification Algorithms

Despite the differences in spectral fluorescence in Figure 2, a device to identify such polymers should be based on algorithms to extract such differences. An example of identification algorithms is explored, but many alternatives could be implemented, including machine learning approaches.

Instead of the intensity values presented in Figure 2, the identification algorithm should be based on ratios between emission intensities. Using ratios of fluorescence intensities offers several significant advantages over using raw intensity values alone, in particular, the normalization of values and the reduction in variability.

Raw fluorescence intensity can be affected by variations, such as fluctuations in LED intensity, photodiode sensitivity, and alignment. Factors like sample thickness, surface roughness, and concentration can influence emission intensity. Temperature changes and ambient light can also affect emission intensity. The use of ratios normalizes these variations, thereby reducing the influence of these variables and providing more consistent and reliable data.

Classification problems often rely on distance-based methods to determine if a data point is unusual or belongs to a specific class. Two of the most common distance measures used are Euclidean distance and Mahalanobis distance.

Euclidean distance is widely used for distance measurement in classification problems and algorithms such as k-Nearest Neighbors (k-NN) and clustering. It measures the “straight-line” distance between two places in space, which is useful in scenarios when the geographic closeness of the data points is significant. The distance separating two points X and Y is determined using Equation (1).

(1) $$D_{E}\left(X,Y\right)=\sqrt{\sum_{i=1}^{n}{\left(x_{i}-y_{i}\right)}^{2}}$$

Mahalanobis distance is a statistic for the distance between a point and a distribution (often the mean of a dataset). In contrast with Euclidean distance, which assumes invariance of all features, Mahalanobis distance uses set variables as weights determining the importance of a set of features with respect to each other and the distribution of the data. This is useful in determining the location of the specific data point in relation to the center of the distribution defined in terms of the data’s covariance. For the two points X and Y in an n-dimensional space, the Mahalanobis distance $D_{M}\left(X,Y\right)$ is defined by Equation (2).

(2) $$D_{M}\left(X,Y\right)=\sqrt{{\left(X-\mu \right)}^{T}S^{-1}(X-\mu )}$$

where *µ* is the mean of the data (the centroid), $S^{-1}$ is the inverse of the covariant matrix of the dataset, and ${\left(X-\mu \right)}^{T}$ is the transpose of the difference between the point and the mean.

Euclidean distance is simple and works well for independent features, but it does not account for correlations and feature scale differences. Mahalanobis distance is more powerful for complex, high-dimensional data with correlated features and varying variances, as it adjusts for the covariance between features. Euclidean distance will be used, since the use of ratios instead of intensity raw values normalizes scales and minimizes the correlation of variables, and only a sample of each plastic was analyzed, as presented in Figure 2.

A similar method to Euclidean distance is implemented; however, each distance is normalized, since each ratio can have very different values (3).

(3) $$D_{E}\left(X,Y\right)=\sqrt{\sum_{i=1}^{n}{\left(\frac{x_{i}-y_{i}}{y_{i}}\right)}^{2}}$$

where $x_{i}$ is the value obtained for each ratio in an unknown plastic sample, and $y_{i}$ is a reference ratio from Table 3; the index I is the line number of Table 3.

The algorithm implements the following steps:

1. For an unknown sample, read the intensity of each photodetector for each excitation.
2. Calculate the 23 ratios between emission intensities as in Table 3.
3. Calculate the six distances between the unknown sample and each of the six references of Table 3, using Equation (3).
4. The smallest distance corresponds to the identified plastic.

## 4. Discussion

The reliability of the classification algorithm was tested by introducing random variations into the fluorescence spectra. For each polymer, errors were introduced randomly, simulating real-world deviations in spectral data. These errors were generated by applying random variations in fluorescence intensity ratios, with error factors of 1.5, 2, 3, 4, and 6. Each error factor corresponds to a percentage variation in each ratio, as shown in Table 4.

**Figure 3:**
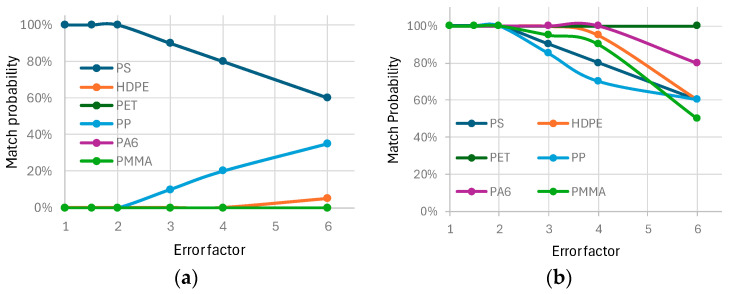
Probability of correct classification of polymers, with error introduced. (**a**) Correct classification probability of a PS polymer and probabilities of false identifications from the others polymers. (**b**) Probability of correct classification of all tested polymers.

For each polymer type and error factor, 20 spectra were generated, resulting in a total of 600 simulated spectra. These were fed into the classification algorithm, and the resulting classifications were compared against the expected polymer types to evaluate accuracy. Figure 3 summarizes the robustness of the classification algorithm.

Figure 3 summarizes the robustness of the classification algorithm. In Figure 3a, the probability of correctly classifying PS is plotted against the error factor, with false identification probabilities for other polymers. Figure 3b presents the overall classification accuracy for all tested polymers. For error factors below 2, corresponding to intensity ratios variations in the range −50% to +100%, all classifications were 100% correct. Even with an error factor of 6, where variations in ratios ranged from −83% to +500%, the probability of correct classification remained above 50%, demonstrating the robustness of the approach.

## 5. Conclusions

This study highlights the effectiveness of a fluorescence-based lab-on-a-chip sensor for polymer identification, offering a compact, low-cost, and efficient alternative to traditional spectroscopy techniques. The suggested system can identify six polymers through the analysis of fluorescence emission patterns under controlled UV excitation, making it highly relevant for applications in recycling, quality control, and environmental monitoring, particularly for microplastic detection.

A lab-on-a-chip device for microplastics detection is proposed using UV LEDs at 265 nm, 285 nm, and 355 nm and five photodetectors with optical filters from 305 to 455 nm.

While passing through the channel, each plastic particle is exposed to UV light, which causes fluorescence. The subsequent fluorescence is then captured and quantified by the photodetectors.

Experimental studies show that the ratios of fluorescence intensities are a robust method for polymer differentiation. The classification algorithm guarantees high accuracy despite changes in the spectral data. The sensor design permits real-time identification and can be used in automated sorting and monitoring systems.

Although the method has been proven to work, it must overcome problems like fluctuations in polymer composition, environmental aging, and additive masking. Further studies should mitigate these problems by broadening the reference database, improving the detection algorithms, and utilizing sophisticated data processing methods like Principal Component Analysis (PCA) and machine learning for better classification.

Overall, the research undertaken represents an essential milestone toward practical and wide-ranging fluorescence-based polymer identification systems. As this technology undergoes further refinement and verification, it stands to improve the efficiency of material separation, advance recycling efforts, and bolster environmental oversight in relation to plastic pollution.

Additionally, the proposed sensor concept may offer valuable applications in the biomedical field, particularly in the analysis of suspensions containing sterically stabilized polymer or polymer-coated magnetic particles. The lab-on-a-chip architecture, combined with real-time fluorescence analysis, could support the development of compact and portable biomedical characterization tools [17]. Furthermore, the integration of magnetic detection techniques into the current optical-based system may lead to the creation of a multifunctional sensor capable of simultaneously analyzing magnetic and optical properties [18]. Incorporating such capabilities into future versions of the sensor could significantly broaden its range of applications, including the detection of magneto-polymer composites or tagged biological targets in flow.

In future undertakings, the authors intend to deploy the system and validate it against a wide array of polymer samples.

## Figures and Tables

**Table 1:** Melting points and thermal press temperatures for polymer film production.

Polymer	Melting Point	Thermal Press Plate Temperature
PET	260°	280°
HDPE	130°	245°
PA6	220°	245°
PMMA	160°	230°
PP	160°	230°
PS	235°	220°

**Table 2:** Optoelectronic components for sensor development.

	Characteristics	Reference
Excitation LED	265 nm	SU CZHEF1.VC-U1U2-L0-V2
	285 nm	SML-LXF3535UVCC10
	355 nm	NDU1104ESE-365-TR
Optical Filters (12.5 mm)	300 nm	300 nm CWL, 12.5 Dia. Hard Coated OD 4.0 25 nm Bandpass Filter (Edmund Optics)
	355 nm	350 nm CWL, 12.5 Dia. Hard Coated OD 4.0 25 nm Bandpass Filter (Edmund Optics)
	370 nm	375 nm CWL, 12.5 Dia. Hard Coated OD 4.0 25 nm Bandpass Filter (Edmund Optics)
	405 nm	400 nm CWL, 12.5mm Dia. Hard Coated OD 4.0 25 nm Bandpass Filter (Edmund Optics)
	455 nm	450 nm CWL, 12.5mm Dia. Hard Coated OD 4.0 25 nm Bandpass Filter (Edmund Optics)
Photodetector	190 to 1000 nm Photosensitive area *ϕ* 0.8 mm	S16586 (Hamammatsu)

**Table 3:** Fluorescence emission ratios for different polymers using 265, 285, and 355 nm as excitation and 305, 355, 370, 405, and 455 nm as emission.

Excitation (nm)	Emission (nm)		Ratio	PS	HDPE	PA6	PMMA	PET	PP
265	305	355	I(305)/I(355)	0.798	0.990	0.253	0.917	0.050	2.121
265	305	370	I(305)/I(370)	1.401	1.138	0.166	0.789	0.033	2.933
265	305	405	I(305)/I(405)	6.727	2.921	0.088	0.812	0.047	8.908
265	305	455	I(305)/I(455)	23.02	7.472	0.106	1.155	0.210	22.07
265	355	370	I(355)/I(370)	1.757	1.150	0.655	0.860	0.667	1.383
265	355	405	I(355)/I(405)	8.434	2.950	0.346	0.885	0.949	4.200
265	405	370	I(405)/I(370)	0.208	0.390	1.896	0.972	0.702	0.329
265	455	355	I(455)/I(355)	0.035	0.133	2.389	0.794	0.238	0.096
265	455	370	I(455)/I(370)	0.061	0.152	1.565	0.683	0.159	0.133
265	455	405	I(455)/I(405)	0.292	0.391	0.826	0.702	0.226	0.404
285	305	355	I(305)/I(355)	2.159	0.226	0.366	0.333	0.038	1.122
285	305	370	I(305)/I(370)	3.158	0.329	0.254	0.300	0.025	1.886
285	305	405	I(305)/I(405)	9.727	0.930	0.166	0.376	0.035	6.161
285	305	455	I(305)/I(455)	37.80	1.848	0.235	0.623	0.151	12.15
285	355	370	I(355)/I(370)	1.463	1.453	0.693	0.900	0.667	1.681
285	355	405	I(355)/I(405)	4.505	4.110	0.452	1.129	0.930	5.492
285	405	370	I(405)/I(370)	0.325	0.353	1.535	0.798	0.717	0.306
285	455	355	I(455)/I(355)	0.057	0.122	1.560	0.535	0.249	0.092
285	455	370	I(455)/I(370)	0.084	0.178	1.082	0.482	0.166	0.155
285	455	405	I(455)/I(405)	0.257	0.503	0.705	0.604	0.232	0.507
355	405	370	I(405)/I(370)	2.096	3.952	5.091	2.521	1.141	3.519
355	455	370	I(455)/I(370)	1.451	3.183	4.173	1.721	0.343	2.492
355	455	405	I(455)/I(405)	0.692	0.806	0.820	0.683	0.301	0.708

**Table 4:** Ranges of random errors introduced in each ratio for robustness analysis.

Error Factor	Maximum Decrease	Maximum Increase
1.5	−33%	+50%
2	−50%	+100%
3	−67%	+200%
4	−75%	+300%
6	−83%	+500%

## Data Availability

The original contributions presented in this study are included in the article. Further inquiries can be directed to the corresponding author.

## Abbreviations

The following abbreviations are used in this manuscript:

EEM	Excitation–Emission Matrix
HDPE	High-Density Polyethylene
PA6	Polyamide 6
PDMS	Polydimethylsiloxane
PET	Polyethylene Terephthalate
PMMA	Polymethyl Methacrylate
PP	Polypropylene
PS	Polystyrene
UV	Ultraviolet
